# Exercise for osteoporosis: A literature review of pathology and mechanism

**DOI:** 10.3389/fimmu.2022.1005665

**Published:** 2022-09-09

**Authors:** Lin Zhang, Yi-Li Zheng, Rui Wang, Xue-Qiang Wang, Hao Zhang

**Affiliations:** ^1^ Department of Sport Rehabilitation, Shanghai University of Sport, Shanghai, China; ^2^ Department of Rehabilitation Medicine, Shanghai Shangti Orthopaedic Hospital, Shanghai, China; ^3^ Department of Orthopedics, Changhai Hospital Affiliated to the Navy Military Medical University, Shanghai, China

**Keywords:** osteoporosis, exercise, inflammatory reaction, cytokines, mechanical stress

## Abstract

Osteoporosis (OP) is a disease that weakens bones and has a high morbidity rate worldwide, which is prevalent among the elderly, particularly, women of postmenopausal age. The dynamic balance between bone formation and resorption is necessary for normal bone metabolism. Many factors, including aging, estrogen deficiency, and prolonged immobilization, disrupt normal apoptosis, autophagy, and inflammation, leading to abnormal activation of osteoclasts, which gradually overwhelm bone formation by bone resorption. Moderate exercise as an effective non-drug treatment helps increase bone formation and helps relieve OP. The possible mechanisms are that exercise affects apoptosis and autophagy through the release of exercise-stimulated myohormone and the secretion of anti-inflammatory cytokines *via* mechanical force. In addition, exercise may also have an impact on the epigenetic processes involved in bone metabolism. Mechanical stimulation promotes bone marrow mesenchymal stem cells (BMSCs) to osteogenic differentiation by altering the expression of non-coding RNAs. Besides, by reducing DNA methylation, the mechanical stimulus can also alter the epigenetic status of osteogenic genes and show associated increased expression. In this review, we reviewed the possible pathological mechanisms of OP and summarized the effects of exercise on bone metabolism, and the mechanisms by which exercise alleviates the progression of OP, to provide a reference for the prevention and treatment of OP.

## Introduction

Osteoporosis (OP) refers to a skeletal disease characterized by low bone density and microarchitectural deterioration of bone tissue consequently increasing bone fragility and susceptibility to fracture as defined by the World Health Organization. OP causes more than 8.9 million fractures annually worldwide, resulting in an osteoporotic fracture every 3 seconds (1). One in three women and one in five men of age over 50 years will experience osteoporotic fractures (2).

OP can occur due to many factors, including senility, reduction in mechanical stimulation, bone and hormone metabolism disorders, and varying properties of stress associated with transcriptional changes in osteogenic genes (3). These factors can lead to a disturbance in the dynamic balance between bone formation dominated by osteoblasts (OBs) and bone resorption dominated by osteoclasts (OCs). In clinical practice, drug therapy such as calcium, vitamin D, hormone replacement therapy, bisphosphonates, etc., are commonly used to treat OP. However, drug therapy has disadvantages such as a long treatment cycle, various drug side effects, high treatment cost, and low compliance (4). Therefore, exercise therapy has attracted increasing attention because of its few adverse reactions, high safety, practicality, and simple operation.

Rahimi et al. (5) conducted a meta-analysis, including 16 randomized controlled trials (RCTs), that summarized the curative efficacy of different training patterns on bone mineral density (BMD) in postmenopausal women. There was no crucial change in BMD of the lumbar spine or femoral neck after exercise training. However, subgroup analysis by exercise training type showed that lumbar BMD was significantly higher when whole-body vibration (WBV) was used as an intervention than with randomized controlled trials with aerobic, resistance, and combined training. This suggests that WBV is an effective method for improving lumbar BMD among elderly postmenopausal patients. In addition, another meta-analysis of 97 RCTs showed that in patients with OP or osteopenia, mind-body exercises (such as Tai Chi, yoga, and dancing) could improve BMD in the lumbar vertebra and femoral neck. Meanwhile, resistance training was more likely to improve BMD in the total hip (6).

However, there was no review on the effect of exercise on the pathological mechanism of OP. Therefore, this review mainly summarizes the possible pathological mechanisms of OP and the mechanisms of exercise alleviating the progression of OP, to provide a reference for the prevention and treatment of OP.

## Pathological changes mechanism of OP

**Figure 1:**
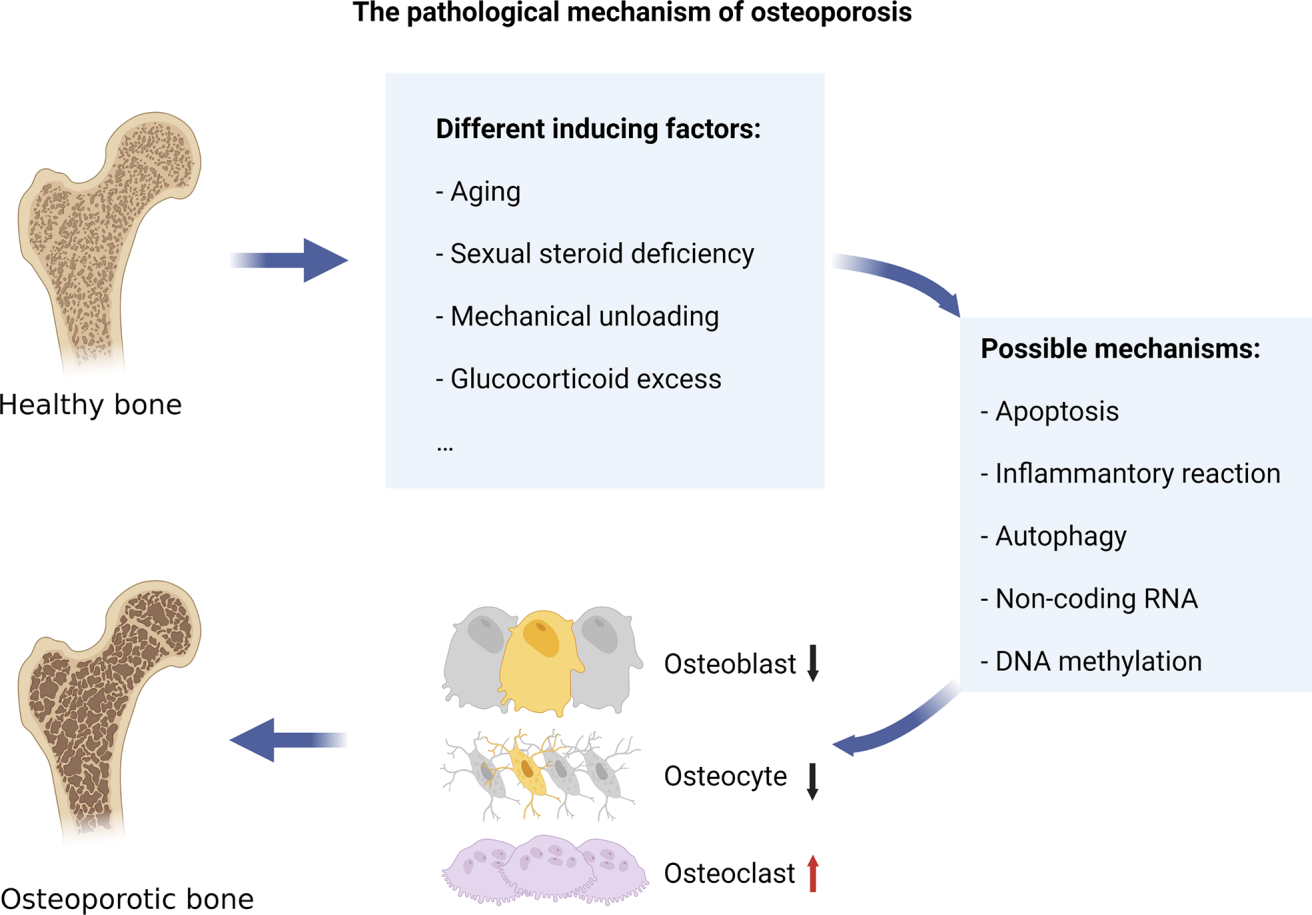
The pathological mechanism of osteoporosis. Various internal and external factors, such as aging, sexual steroid deficiency, mechanical unloading, and overuse of glucocorticoids, can cause bone resorption to exceed bone formation, leading to osteoporosis. The pathological mechanisms of OP mainly include the abnormal activation of osteoclasts resulting from changes in apoptosis, inflammatory reaction, and autophagy. Besides, epigenetic changes, such as changes in non-coding RNA and DNA methylation, can also reduce the expression of osteogenic genes.

When osteoclastogenesis exceeds osteoblastogenesis, the resulting condition is an OP (3). Bone metabolism is in a state of dynamic balance, which is jointly maintained by bone formation led by OBs and bone resorption led by OCs (coupling between OBs and OCs). However, when osteoclast-dominated bone resorption activity is enhanced and if there is no corresponding bone formation activity, it leads to BMD loss and even OP. Estrogen levels in postmenopausal women drop because of ovarian failure. Estrogen deficiency increases bone turnover and imbalances in bone metabolism by affecting levels of T lymphocyte, B lymphocyte, monocytes, and cytokines (7). This leads to increased BMD loss, destruction of trabecular microstructure, and increased risk of fracture. The pathological mechanisms of OP mainly include the abnormal activation of OCs resulting from changes in apoptosis, inflammatory reaction, and autophagy. Besides, epigenetic changes, such as changes in non-coding RNA and DNA methylation, can also reduce the expression of osteogenic genes (**Figure 1**).

### Apoptosis

Apoptosis is a normal physiological process. It refers to the process of cell death caused by internal and external factors triggering the preexisting intracellular death process. However, excessive or defects of apoptosis can lead to bone-related diseases (8). Some studies have shown that the maintenance of BMD depends not only on the absorption function of OCs and the osteogenic function of OBs but also on the changes in the lifespan of the two kinds of cells by apoptosis (9). Osteocytes, derived from OBs, are the major cells in mature bone tissue, and they can survive for decades (9).

Evidence obtained from studies conducted in mice illustrates that increased osteocyte apoptosis is partly responsible for osteoporosis caused by sex steroid deficiency, overuse of glucocorticoids (GCs), or senescence (10). The upregulated expressions of receptor activator of nuclear factor κ-B ligand (RANKL) and vascular endothelial growth factor (VEGF) in aged mice may inhibit osteoblast maturation or induce osteocyte death (11). By increasing the signal of Ras/Rac1/Erk and PI3 kinase/mTOR/S6K, cytokines such as macrophage colony-stimulating factor (M-CSF), RANKL, tumor necrosis factor (TNF), and VEGF can slow down apoptosis and improve the survival rate of OCs, which is linked to the rise in bone resorption (12). Weinstein and Manolagas (9) found that osteoblast apoptosis increased 10-fold, meanwhile, osteoblast apoptosis increased fourfold in the vertebrae of ovariectomized mice as compared with the control group. This indicated that the acceleration of bone loss after the lack of estrogen is caused by an earlier reduction in the working time of OBs. Hughes et al. (13) reported that 17β-estradiol increased the apoptosis rate of rat OCs from 0.5% to 2.7%, and estrogen deficiency prolonged the survival period of OCs, increasing the number of cells by 2–3 times, and leading to enlargement of the bone absorption cavity. In addition, OBs, as the main target of GCs, play an important role in the pathogenesis of GC-induced OP. GCs can increase the expression of BH3-only protein Bim and downregulate tissue inhibitor of metalloproteinase-1 (TIMP-1) to promote osteoblast apoptosis, thereby leading to the occurrence of OP (14, 15). The apoptosis of osteocytes and OBs in GC-induced OP is caused by the loss of extracellular matrix attachment mediated by the internal and external signaling pathways of Pyk2/JNK kinase (16). GCs, increase reactive oxygen species (ROS) production *in vivo* bone and *in vitro* OBs (17), which will promote the activation of a PKCβ/p66(shc)/JNK signaling cascade.

In addition, mechanical unloading, such as bed rest, reduced activity during senescence, or space travel, frequently causes BMD loss. Aguirre et al. (18) found that increased apoptosis of osteocytes was directly affected by mechanical unloading through tail suspension mice models. Although reducing osteoblast apoptosis can raise BMD, p53 mostly controls how apoptosis affects bone formation. Preventing osteoblast proliferation and apoptosis and encouraging osteoblast differentiation *via* the Akt-FoxOS pathway, has the opposite effect on the development of OCs (11). Connexin43 (Cx43) expression in the bone can decline with age, and Cx43 knockout increases osteocyte apoptosis, the number of OCs, and bone resorption on the cortical bone surface (19). In addition, Davis et al. identified a brand-new Cx43/miR21/HMGB1/RANKL pathway that controls osteoclast production and recruitment, is decreased with aging, and is important in preventing osteocyte apoptosis.

### Inflammatory reaction

A higher incidence of various age-related illnesses, including OP and fragile fractures, is linked to chronic inflammation (20). Osteoclast-mediated bone resorption is accelerated by pro-inflammatory signals acting on mesenchymal stem cells and osteoclast precursors. (21). Age-related BMD loss and increased bone resorption are predicted by higher serum inflammatory marker levels in older adults (22). According to research by Cauley et al. (20), high serum levels of inflammatory markers in older adults who were in good health indicated a greater risk of fractures during a 5.8-year follow-up period.

The immune system’s role in the development of senile OP arises mainly through the effects of estrogen deficiency and secondary hyperparathyroidism. In addition to directly affecting bone cells, the significant postmenopausal estrogen deficiency among postmenopausal women, indirectly causes them to exhibit chronic low-grade inflammatory phenotypes, namely altered cytokine expression and altered immune cell morphology (23). According to studies, a lack of estrogen led to a large rise in the pro-inflammatory cytokines, which include interleukin-1(IL-1), interleukin-6(IL-6), TNF-α, M-CSF, and prostaglandin E2(PGE2) (24–26). Most of these cytokines, directly and indirectly, act on OBs and OCs, improve the differentiation of mononuclear cells to mature OCs, and regulate osteoclast function. TNF-α can increase osteoblast apoptosis and the expression of RANKL. This indirectly leads to the increase of osteoclast differentiation and activity and inhibits osteoclast apoptosis (27), thus leading to BMD loss in the postmenopausal OP. Zha et al. (28) found that postmenopausal women with OP (PMOP) had higher levels of TNF-α than those without the condition. By stimulating the NF-κB and PI3K/Akt pathway *in vitro*, TNF-α synergistically enhances RANKL-induced osteoclast formation. Some of these cytokines, like IL-1 and TNF-α, may interact with one another in a way that promotes increased production of the other, which would further stimulate bone resorption. While PGE2 raises RANKL and lowers OPG, IL-1 and TNF-α enhance RANKL, OPG, and M-CSF (25, 29).

In addition, estrogen affects the proliferation, differentiation, activation, and homing of various immune cells (23). Various immune cells directly or indirectly affect bone cells through other mediators secreted by immune cells such as OPG/RANKL, and inflammatory cytokines such as IL-6 and TNF-α. The signaling pathways for NF-κB and c-src/PI 3-kinase/Akt are started when these immune factors activate TNF-related factors (TRAFs). Activating downstream molecules like c-Fos and Fos B causes the nuclear factor of activated T-cells cytoplasmic 1 (NFATc1) to be produced, which in turn causes the maturation and activation of osteoclasts (30). An immunoclinical study shows that CD19+ B lymphocytes are less abundant in postmenopausal women, however, they secrete more granulocyte-macrophage-colony-stimulating factor (GM-CSF) (31). Another study shows that T cells are more likely to express TNF-α in postmenopausal OP patients with bone fractures (32). Two recent studies have shown an increase in circulating T cells and monocytes in postmenopausal women (33) and a higher neutrophil-to-lymphocyte ratio in their peripheral blood (34).

### Autophagy

Autophagy is a highly conserved cellular behavior, in which cells enfold intracytoplasmic substrates, such as senescence proteins, organelles, misfolded proteins, and damaged mitochondria, into autophagy by forming a double-layer membrane structure, and transport the substrates to the lysosome for degradation and then release them back to the cytoplasm (35). Autophagy can maintain cell activities and play a protective role through material recycling under the conditions of cell starvation, hypoxia, lack of growth factors, and some pathological conditions (36). The autophagy-associated gene (ATG) family of proteins regulates the formation of autophagosomes, which is the first step at the beginning of autophagy (37). A genome-wide association study confirmed the correlation between genetic variation of several ATG proteins and wrist BMD. It suggested that autophagy alteration may lead to OP phenotype (38).

Age-related autophagy suppression or decreased efficiency might result in BMD loss among the elderly population (39, 40). In addition, an important risk factor for OP associated with aging is vitamin D (VD) insufficiency. VD3 raises cytoplasmic calcium levels, which promotes autophagy. As a result, the CaMKK-β-AMPK signaling pathway is activated, which inhibits the activity of mTORC1 (41). Increased GC levels are another factor that leads to aging and excessive GCs can induce bone marrow mesenchymal stem cells (BMSCs) apoptosis. By inducing autophagy, GC-induced apoptosis in BMSCs may be prevented (37).

Reduced levels of osteoblast autophagy have been reported in rat models of OP (42). Mice with osteoblast-specific deletion of FIP200, a key element of mammalian autophagy, develop osteopenia as a result of OBs’ defective terminal differentiation and a reduction in bone production (43). Onal et al. (44) conditionally knocked out the osteocyte’s autophagy gene Atg7 in 6-month-old mice. In addition to a decline in the quantity of OCs and OBs, a decline in bone production rates, and an increase in oxidative stress, they discovered a drop in bone mass. These changes are also characteristics of aging mice. In addition, autophagy inhibits osteoblast apoptosis mediated by oxidative stress (45). Liu et al. (46) identified the autophagy receptor Optineurin (OPTN) as a key molecule to determine the cell fate of BMSCs, and its expression was decreased in aged mice. Aged mice and optn ^-/-^ mice showed an osteoporotic bone loss, increased aging of BMSCs, decreased osteogenesis, and increased adipogenesis. On the contrary, the inactivation of mouse monocyte autophagy-associated gene 7 (Atg7) prevented osteoclast differentiation while mitigating bone loss in mice treated with glucocorticoid or ovariectomized (47). Xiu et al. (48) discovered that RelB, a member of the NF-κB family, can cause TRAF3 degradation by controlling the transcription of BECN1, an early autophagy protein, in relation to the impact of autophagy on osteoclasts. The NF-κB signaling pathway is activated by TRAF3 degradation, which encourages RANKL-induced osteoclast development.

### Changes in non-coding RNA

Non-coding RNA, a group of functional RNA not encoding proteins, plays a role in the occurrence and progression of several diseases, including OP. According to one study, postmenopausal OP patients (OP group) had differing levels of expression from healthy controls for 13 microRNAs (miRs), 70 long non-coding RNAs (lncRNAs), and 260 circular RNAs (circRNAs) (49).

LncRNAs with lengths longer than 200 nucleotides are transcripts that are not translated into protein (50). Although lncRNA does not encode proteins, it helps in regulating gene transcription in two ways: trans and cis (49). OP patients and ovariectomized mice had higher levels of lncRNA MEG3 in their BMSCs, which suppresses osteogenic development by downregulating miR-133a-3p (51). In response to mechanical stimulation, Liu et al. (52) demonstrated that the lncRNA Neat1 regulates osteoblast activity and bone formation through paraspeckle-dependent E3 ubiquitin ligase Smurf1 mRNA nuclear retention. Following disruption of paraspeckles, Neat1 was deficient, which resulted in lower osteoblast activity and decreased BMD. The alterations in bone formation in response to mechanical loading and unloading were reduced in mice models with Neat1 depletion.

Because their many target mRNA transcripts are involved in cell proliferation, differentiation, and death, miRs play a role in determining the determination of a cell’s fate. Chen et al. (53) found that the reduced expression of miR-503 in CD14 + peripheral blood mononuclear cells (PMBCs) may be one of the pathogenesis of postmenopausal OP patients because miR-503 can specifically bind to RANKL, which can suppress the formation of OCs. Sugatani et al. (54) demonstrated that miR-21 high expression can stimulate osteoclast generation by downregulating levels of programmed cell death 4 (PDCD4), a protein that acts as a suppressive regulator of osteoclast production.

CircRNAs are another non-coding RNA mainly found in the cytoplasm in mammalian cells. They function as both indicators for various illnesses and essential components in tissue growth. Huang et al. (55) discovered that during osteoblast generation induced by recombinant NELL‐1, the expression of circRFWD2 and circINO8 increased and was inversely linked with hsa‐miR‐6817‐5p. In BMSCs from nonunion patients, Ouyang et al. (56) examined the differential expression of circRNAs and discovered that hsa_circ_0074834 had a much lower expression level. By controlling the expression of f Zeb1 and VEGF through miR-942-5p, has_circ_0074834 encourages BMSCs to differentiate into osteogenic tissue.

### DNA methylation

The process of DNA methylation, one of the most durable epigenetic changes of gene transcription, is carried out by the DNA methyltransferase family (DNMT), which adds a methyl group to the cytosine base’s five carbon position (57, 58). Increased levels of DNA methylation can suppress gene expression, possibly through transcriptional repression by recruiting proteins that prevent transcription factors from binding to DNA (59). Recent research has demonstrated that aberrant epigenetic alteration, which may result from both individual genetic variables and environmental triggers, is connected with a homeostatic imbalance between bone creation and resorption (60, 61). Reppe et al. (62) revealed an association between DNA methylation and BMD and fracture risk in postmenopausal women.

Raje et al. (63) compared CpG methylation levels of BMP2 promoters in OP patients and healthy people. They found that transcriptional activity and gene expression of BMP2 promoters in OP patients were reduced, which could downregulate the osteoblast markers involved in bone formation. According to Chen et al. (3), abnormal DNA methylation changes resulted in diminished femoral nuclear factor E2-related factor 2 (Nrf2), which is directly related to the development of OP. The simultaneous increase of DNA methyltransferase (Dnmt)1/Dnmt3a/Dnmt3b and hypermethylation of the Nrf2 promoter dramatically suppressed femur Nrf2 in OP patients and ovariectomized mice. In addition, Dnmt3a also has a significant impact on the abnormal activation of OCs. Nishikawa et al. (64) found that, through epigenetic inhibitions of the IRF8 (a negative regulator of osteoclast-induced differentiation) gene, RANKL-induced activation of Dnmt3a can mediate DNA methylation *via* S-adenosylmethionine (SAM), this promotes osteoclast formation. Moller et al. (65) induced the differentiation of blood CD14+ monocytes into OCs from women of different ages and menopausal states and found that older women also had higher bone resorption activity of differentiated OCs *in vitro*, which may be related to the decreased DNA methylation of the TM7SF4 promoter, a key OCs gene.

## Mechanisms of exercise in alleviating OP

Regular exercise helps keep the bone healthy and improve bone health by stimulating bone formation and strength through mechanical loading, even if it does not significantly improve BMD (66). Skeletal unloading occurs to some extent as people age due to decreases in physical activity and increases in sedentary time (67). From a mechanically-centric point of view, activities that generate higher intensity or quicker loads (such as resistance training and leaping) are excellent for promoting bone health because they stimulate existing bone cells in a significant way. Consequently, bone-healthy exercise encourages mesenchymal stem cells to differentiate into osteoblast lineages, thereby producing more healthy bone cells (68). As described by Robling et al. (69), mechanical forces applied to bone tissue induce the movement of interstitial fluid along tubules and bone cell pores, thereby causing cell-level shear stress and deformation of bone cell plasma membrane. These changes lead to the beginning of the bone remodeling process and stimulate bone resorption and formation cycles (70). Studies have shown that weight-bearing exercise, resistance training, or WBV training help maintain or improve bone mass, and improve the BMD of postmenopausal women, thereby promoting health and improved quality of life (71–73).

In addition, independent of mechanical loads, aerobic exercise can enhance osteocyte survival through altered macronutrient transport, the release of exercise-stimulated myohormones, and preservation of cellular or mitochondrial repair (68). The likely reason is that muscle factors (such as myostatin and irisin) secreted by muscle and bone factors (such as osteocalcin, TGF-β, and PGE2) secreted by osteocytes can interact with each other, and their secretion is regulated by mechanical load (74). Other studies have shown that by enhancing aerobic glycolysis, irisin can promote osteoblast proliferation (75). Several studies have shown that regular endurance exercise can slow the age-related degeneration of mitochondrial number and capacity and dysfunction (76, 77).

In addition, proper exercise helps improve balance and postural stability (e.g., Tai Chi and Yoga) and reduces the frequency of falls, thus effectively reducing the occurrence of OP fractures thereby protecting the bone from trauma (Turner et al., 2003). A meta-analysis has shown that Tai Chi practice can reduce the risk of falls and fall-related injuries by approximately 43% and 50% in the short term (< 12 months) in at-risk adults and older adults, respectively (78). Moreover, a 12-week Iyengar yoga program can significantly improve standing balance by conducting sit-to-stand tests and a 4-minute walk for elderly community residents as compared to the control group (79).

**Figure 2:**
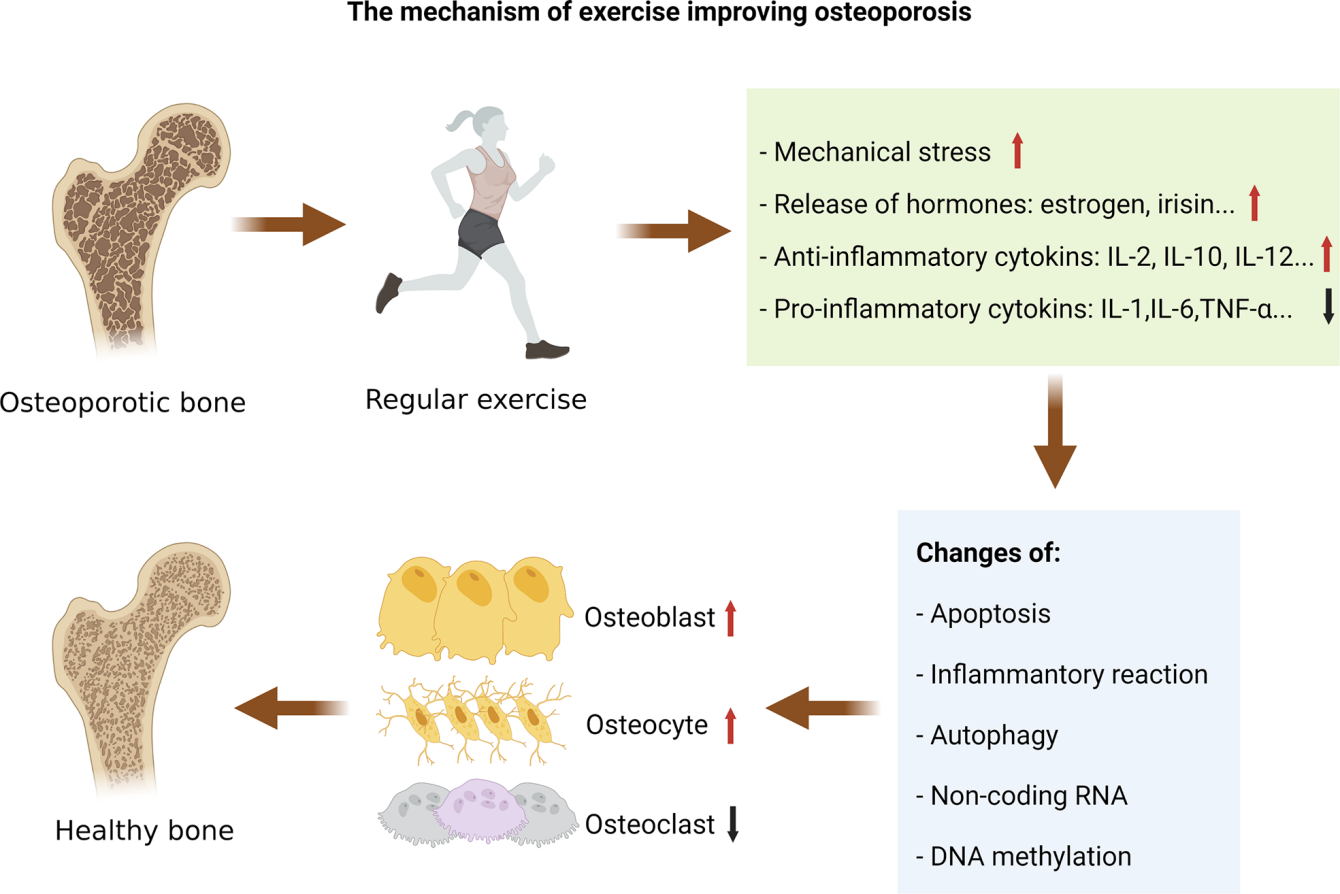
The mechanism of exercise improving osteoporosis. Exercise-induced changes in mechanical stress, hormones, and cytokines can regulate the pathological changes of osteoporosis. Mechanistically, exercise reduces the harmful osteoporosis alterations *via* affecting apoptosis, inflammatory response, and autophagy, and exercise may affect the epigenetic mechanisms of bone metabolism by regulating non-coding RNAs and DNA methylation.

This review summarizes the pathogenic mechanisms of exercise in the therapy of OP in rodents by summarizing the related literature (**Table 1**), and different types of exercise can improve human OP (**Table 2**). Mechanistically, exercise reduces the harmful OP alterations *via* affecting apoptosis, inflammatory response, and autophagy, and exercise may affect the epigenetic mechanisms of bone metabolism by regulating non-coding RNAs and DNA methylation (**Figure 2**).

**Table 1:** Mechanism of exercise in the treatment of osteoporosis (OP) in rodents.

Authors	Model	Exercise types	Related gene/cytokines/protein	Involved in pathways	Improved organization	Functions	Change
Chen et al. (3)	OP mice model	Running exercise	Nrf2, Dnmt1/3a/3b, SOD	Keap1-Nrf2	Femur bone mass and trabecular microstructure	DNA Methylation	↓
Aveline et al. (80)	OP rat model	Jumping exercise	Caspase-3	–	Whole body BMC and BMD, femur trabecular bone, and cortical microarchitecture	Osteocyte Apoptosis	↓
Maurel et al. (81)	OP rat model	Treadmill training	Caspase-3	–	Femur trabecular microstructure	Osteocyte Apoptosis	↓
Wen et al. (82)	Aged rat model	Low magnitude vibration	P53, P21	Sirt1/p53/p21	Femur BMD and trabecular microstructure	Apoptosis	↓
Li et al. (83)	OP rat model	Running	IL-1b, IL-6, Cox-2	–	Tibias trabecular microstructure	Inflammation	↓
Gao et al. (84)	Female rat model	Treadmill training	serum corticosterone, cortisol, pregnenolone, and estradiol	BDNF/AKT	–	Inflammation	↓
Lee et al. (85)	Middle-aged mice model	Treadmill training	–	–	Femur and tibias BMD and trabecular microstructure and skeletal nerve fiber density	miRNA	↓↑
Liu et al. (52)	OP mice model	Treadmill training	OCN, Col1α1, PINP	–	Femur bone mass and thickness of cortical bone and trabecular microstructure	lncRNA Neat1	↑
Zuo et al. (86)	OP cell model	Mechanical stretch	Runx2	–	Osteoblast differentiation and bone formation	miR-103a	↓

↑: increase; ↓: decrease; ↑↓: The expression of some miRNAs was increased, and the expression of some miRNAs was decreased.

**Table 2:** Different exercise types on human osteoporosis (OP).

Authors		Participants	Duration	Intervention group	Control group	Outcome	Exercise effect
FILIPOVIĆ et al. (87)		Postmenopausal OP women	12 weeks	N = 47 Resistance training, balance exercise, and aerobic exercise	N = 49 No train	TUG, STS, and OLST	Improved the TUG, STS, and OLST.
Hettchen et al. (88)		Postmenopausal OP women	13 months	N = 27 High impact weight-bearing/high-intensity/velocity resistance training	N = 27 Low-intensity exercise	Lumbar spine BMD and total hip BMD	Improved the lumbar spine BMD.
Kistler Fischbacher et al. (89)		Postmenopausal women with low bone mass	8 months	N = 15 HiRIT-med, N = 14 BB-med	N = 42 HiRIT N = 44 BB	Lumbar spine BMD and total hip BMD	HiRIT improved the lumbar spine BMD more than BB. Antiresorptive medication may enhance exercise efficacy at the proximal femur and lumbar spine.
Stanghelle et al. (90)		65+ years old women diagnosed with OP and vertebral fracture	12 weeks	N = 76 Multicomponent resistance and balance exercise programme	N = 73 No train	Habitual walking speed, physical fitness, health-related quality of life, and fear of falling	Improved muscle strength, and balance and reduce fear of falling.
Stanghelle et al. (91)		65+ years old women diagnosed with OP and vertebral fracture	12 weeks	N = 76 Multicomponent resistance and balance exercise programme	N = 73 No train	Habitual walking speed, physical fitness, health-related quality of life, and fear of falling	Improved muscle strength, balance, and mobility and reduces fear of falling 3 months post-intervention.
Kemmler et al. (92)		Sedentary community-dwelling older men with osteopenia/OP and SMI-based sarcopenia	12 months	N = 21 low-volume/HIT-DRT, with whey protein, VD, and calcium	N = 22 No train, with whey protein, VD, and calcium	Lumbar spine BMD, SMI, total hip BMD, maximum isokinetic hip-/leg-extensor strength	Improved lumbar spine BMD, SMI, and maximum isokinetic hip-/leg-extensor strength.
Harding et al. (93)		Older men with low hip and/or lumbar spine BMD	8 months	N = 34 HiRIT. N = 33 Machine-based IAC	N = 26 No train	Lumbar spine and hip BMD, calcaneal ultrasound parameters, anthropometry, body composition, function (TUG, FTSTS, BES, LES)	Compared with CON, HiRIT improved trochanteric BMD, lumbar spine BMD, BUA, stiffness index, lean mass, TUG, FTSTS, BES, and LES. Compared with CON, IAC improved lean mass and FTSTS. Compared with IAC, HiRIT improved lumbar spine BMD, stiffness index, and FTSTS.
Filipović et al. (94)		Postmenopausal osteoporotic patients	12 weeks	N = 37 Aerobic exercise, resistance training, and balance exercise, with alendronate therapy	N = 31 No train, with alendronate therapy	Activities of serum MMP-9 and TIMP-1	Exercises decreased the activity of serum MMP-9 and increased the activity of TIMP-1.
Harding et al. (95)		Men with low lumbar spine and/or proximal femur BMD	8 months	N = 34 HiRIT N = 33 Machine-based IAC	N = 26 No train	Femoral neck and total hip BMC, volume, and vBMD for total, trabecular, and cortical bone compartments, total femoral neck cortical thickness, geometric and bone structural strength indices	Compared with IAC, HiRIT improved medial femoral neck cortical thickness. Both HiRIT and IAC preserve bone strength at the distal tibia and distal radius.
ElDeeb et al. (96)		Postmenopausal women with low BMD	24 weeks	N = 25 WBV, with VD and calcium supplementations once daily	N = 24 No train, with VD and calcium supplementations once daily	BMD of the lumbar vertebrae and femur, and hip/knee/ankle muscle work during gait	Improved the leg muscle work and lumbar and femoral BMD.
Sen et al. (97)	Postmenopausal women		6 months	N = 19 WBV N = 19 High impact training, with 1500 mg of calcium and 880 IU of VD per day	N=20 No train, with 1500 mg of calcium and 880 IU of VD per day	BMD of the lumbar spine and femur, serum markers, functional mobility, fall index, HRQoL, and depressive symptoms	WBV can prevent bone loss, and WBV and high impact training can improve functional mobility, HRQoL, and depressive symptoms.
Pérez-Gómez et al. (98)	Postmenopausal women		12 weeks	N = 13 WBV	–	SMP30, body composition, (fat mass, lean mass, and bone mass) physical fitness (balance, TUG, and 6MWT)	Increased SMP30 in plasma, and 6MWT, reduced SMP30 in platelets, TUG, and total body fat mass.
Pasqualini et al. (99)	Postmenopausal women with a T-score at the lumbar spine or femoral neck between - 1 and - 2.5 SD		3 months	N = 33 weight-bearing and resistance exercise	–	Anthropometric and fitness parameters, bone-remodeling markers, OCs, and QoL	Increased the markers of bone formation and the commitment of immature OCs, and improved the score of QoL with pain, physical function, and mental function.

TUG, timed up-and-go; STS, sit to stand test; OLST, one leg stance test; BMD, bone mineral density; HiRIT, high intensity progressive resistance and impact training; BB, Buff Bones^®^, SMI, skeletal muscle mass index; HIT-DRT, high intensity dynamic resistance exercise; VD, vitamin D; IAC, isometric axial compression; FTSTS, five-times sit-to-stand; BES, back extensor strength; LES, leg extensor strength; CON, control; BUA, broadband ultrasound attenuation; BES, back extensor strength; BMC, bone mineral content; vBMD, volumetric bone mineral density; WBV, whole-body vibration; IU, international unit; HRQoL, health-related quality of life; SMP30, Regucalcin or senescence marker protein 30; 6MWT, 6-min walk test; OCs, osteoclasts. ↑: increase; ↓: decrease; ↑↓: The expression of some miRNAs was increased, and the expression of some miRNAs was decreased.

### The role of exercise in apoptosis

One of the processes causing the pathogenic alterations in OP is apoptosis. Aveline et al. (80) measured osteocyte apoptosis as a percentage of caspase-3 immunostaining. They found that ovariectomized rats improved BMD and bone mineral content (BMC), trabecular parameters, cross-sectional area, a moment of inertia and OCN levels, and reduced osteocytes apoptosis and lipid content after 8 weeks of jumping training. In a rat model of secondary OP brought on by excessive alcohol intake, Maurel et al. (81) trained the experimental group on the treadmill. After 14 weeks of training, trabecular and cortical thickness, cortical pores, and osteocyte apoptosis of the rat model were improved.

Wen et al. (82) identified that OBs from old rats all showed senescence changes, among which osteocytes manifest the most evident senescence changes. After low magnitude vibration (LMV) treatment, the expression of anti-aging protein SIRT1 was significantly upregulated, whereas the expressions of p53 and P21 were significantly downregulated. LMV can promote bone formation in aged rats, upregulate the expression of anti-aging protein Sirt1 in osteoblasts, and downregulate the expression of p53 and P21. Thus, LMV can partially inhibit the aging of osteoblasts through Sirt1/p53/P21 axis.

High-intensity exercise raises irisin levels in the blood (100). Irisin is an exercise-related myokine that is crucial for bone remodeling. A study revealed that exercise-mimetic myokine irisin can increase osteocyte functions and exhibits anti-apoptotic effects (101). Irisin’s activation of MAP kinases Erk1 and Erk2 and subsequent upregulation of the transcription factor Atf4 through an Erk-dependent pathway in osteocytes was the underlying mechanism. Moreover, according to Xu et al. (102), irisin reduced the prevalence of postmenopausal OP by upregulating Nrf2, blocking inflammatory bodies containing Pyrin domain protein 3 (NLRP3), and reducing the content of inflammatory factors. These actions together prevented OBs from apoptosis and inhibited osteoblast death. Furthermore, studies have shown that Senescence marker Protein-30 (SMP-30) can protect against cell apoptosis (103), however, the serum level of SMP30 decreases with age in elderly women (104). Pérez-Gómez et al. (98) showed that after 12 weeks of three times-weekly WBV exercise, postmenopausal women’s circulating SMP30 levels, gait efficiency, and fat mass were all improved.

### The role of exercise in inflammatory reaction

Exercise can prevent and reduce OP by reducing pro-inflammatory cytokines and suppressing inflammation. Exercise increases the secretion of anti-inflammatory cytokines like IL-2, IL-10, IL-12, and interferon (IFN), which are beneficial to bone formation, and decreases the secretion of pro-inflammatory cytokines such as IL-1, IL-6, and TNF-α to prevent bone resorption (105). IL-10 is a cytokine that limits the host’s immune response to pathogens and prevents tissue damage. A systematic review discovered a strong correlation between the duration of exercise and the rise in serum IL-10 (106). Santos et al. (107) reported that after 6 months of moderate exercise, older individuals’ quality of life improved with higher serum IL-10 levels and significantly lower IL-6 and TNF-α levels. In addition, according to a meta-analysis, regular exercise reduces IL-6 and C-reactive protein levels among older persons (108). Reducing the expression of monocyte Toll-like receptor (TLR) is one of the potential mechanisms of the anti-inflammatory effects of physical activity. Stewart et al. (109) found that 12 weeks of endurance and resistance exercise can effectively reduce the activation of TLR4 and the generation of pro-inflammatory cytokines IL-6 in previously sedentary older adults and younger adults.

Gao et al. (84) showed that mice that started moderate-intensity continuous training at 8 months had higher levels of serum corticosterone, cortisol, pregnenolone, and estradiol compared to mice that started training at 18 months, and this suggests that reducing aging-related steroid hormone and anti-inflammatory factors is more effectively accomplished by beginning training at 8 months.

### The role of exercise in autophagy

Exercise can regulate bone metabolism by regulating the autophagy of bone tissue cells. Dalle et al. (110) found that physical exercise promoted the differentiation of BMSCs. After a half marathon, the expression of genes associated with telomerase, autophagy, and the genes Runx2, Msx1, and Spp1 was also altered. These changes were positively correlated with the differentiation of BMSCs.

The content of LC3-II, which is the surface marker of autophagic vacuoles, can inadvertently indicate the degree of autophagy (111). Zhang et al. (112) found that fluid shear stress (FSS) causes protective autophagy in osteocytes by inducing autophagic vacuoles, and higher levels of the LC3-II isoform, and the degeneration of P62 in osteocyte-like MLO-Y4 cells. In addition, the survival of osteocytes and ATP metabolism are linked to the autophagy induced by mechanical stimulation.

Treadmill exercise increased AMP-activated protein kinase (AMPK) phosphorylation in mice models, which promotes metabolism for glucose and fatty acid utilization (113). It has been hypothesized that human mesenchymal stem cells’ ability to differentiate into OBs is controlled by AMPK through late activation of the Akt/mTOR signaling axis and early mTOR inhibition-mediated autophagy (114). In addition, irisin increased during and after exercise, may upregulate autophagy by increasing the Atg12-Atg5-Atg16L complex, thereby promoting osteogenesis of BMSCs and enhancing the Wnt/β-catenin signaling pathway (115).

### The role of exercise in non-coding RNA

By controlling the expression of non-coding RNA, exercise can lessen the harmful effects of OP. In the study of An et al. (116), miRs, lncRNAs, and circRNAs of Type 2 Diabetes with Depression (DD) patients were analyzed before and after Baduanjin treatment at 12 weeks. It was confirmed that Baduanjin can effectively improve the depressive symptoms and blood glucose level of DD patients by regulating the abnormal expression of lncRNAs, miRs, and circRNAs. Zhu et al. (117) found that appropriate tensile stress can promote BMSCs to osteogenic differentiation, prevent adipocyte differentiation, and induce the production of lncRNA-MEG3. Furthermore, overexpressed lncRNA-MEG3 further stimulated osteogenic differentiation in stressed BMSCs and inhibited the expression of miR-140-5p. According to Liu et al. (52), OP or unloading-induced bone loss can be prevented by increasing the Neat1 levels or stabilizing the paraspeckle structures by exercise and mechanical stress in conjunction with certain small molecules and oligonucleotides (52). In addition, exercise affects the expression of miRs that control inflammation and triggers changes in the gene expression of neutrophils and peripheral blood mononuclear cells (118). In ovariectomized rats, Li et al. (83) showed that 3-month running exercise can effectively reduce miRs, IL-1, IL-6, and Cox-2 expression levels, inhibit bone resorption, and improve bone trabecular formation. In addition, Zuo et al. (86) identified that *in vivo* and *in vitro* osteogenesis and bone production may be controlled by mechanical stress. Periodic mechanical stretch downregulates miRNA-103A and its host gene PANK3, and promotes the expression of Runx2 protein (a major osteogenic transcription factor), suggesting that the down-regulation of miRNA-103A may be a crucial factor for mechanically stimulating bone growth.

Few research has been done so far on the impact of circRNAs on bone during exercise, however, to understand their purpose, further research is needed to elaborate on their effects. Lee et al. (85) found that improvements in trabecular bone microarchitecture in middle-aged mice were directly associated with increased skeletal nerves after aerobic exercise training. And the expression of seven upstream osteogeneses and neuroplasticity non-coding RNAs, including miR-491-3p, miR-470-5p, let-7a-5p, miR-137-3p, miR-130a3p, and miR-29b-3p, were upregulated after 8 weeks of treadmill activity training. Guo et al. (119) conducted a study and confirmed that the expression of circBBS9 in the quadriceps femoris of the aging mice decreased with age compared to the young mice, however, it was reversed after 2 months of treadmill exercise. Fang et al. (120) found that aerobic exercise helps reduce cartilage tissue damage, inflammatory cytokines content, type II collagen, chondrogenic differentiation-related genes, and circUNK expression induced by knee osteoarthritis.

### The role of exercise in DNA methylation

DNA methylation is one of the known epigenetic mechanisms modulated by exercise (57). When it comes to aging, cancer, and type II diabetes, appropriate physical activity can help to control DNA methylation, as discovered by recent studies (121). A study was conducted involving 12 young men and 11 young women who underwent 3 months of one-legged knee-extension exercise and found that methylation at 4, 919 sites in the leg genome was altered after endurance training as compared with pre-training (122). From 23 healthy men, Rönn et al. (123) analyzed genome-wide patterns of DNA methylation in human adipose tissue. After a 6-month exercise intervention, including spinning and aerobics, global DNA methylation changed and a total of 17, 975 individual CpG sites of 7, 663 distinct genes showed altered DNA methylation levels. These results suggest that exercise can induce genome-wide alternations in DNA methylation in human adipose tissue and may affect the metabolism of adipocytes. Arnsdorf et al. (124) demonstrated that mechanical stimulation alters the epigenetic status of osteogenic genes by reducing DNA methylation and shows associated increased expression. This promotes the migration of MSCs and increases the potential for osteogenic differentiation.

In addition, Chen et al. (3) further demonstrated that running exercise (RE) ameliorated OP by correcting hypermethylation of the anti-osteoporotic factor Nrf2 promoter in an ovariectomized OP mouse model. The possible mechanism is that RE may reduce Nrf2 inhibition by blocking the abnormally elevated Dnmt, which demethylates the Nrf2 promoter. Nakajima et al. (125) investigated the epigenetic effects of exercise and aging on CpG island methylation in the ASC gene, which is involved in IL‐1β and IL‐18 secretion and whose expression increases with age. Age-induced reductions in ASC methylation led to an increase in pro-inflammatory status, however, 6 months of high-intensity intermittent walking reduced age-dependent reductions in ASC methylation and inhibited excess pro-inflammatory cytokines by reducing ASC expression.

## Conclusion and outlook

This review describes that the changes in apoptosis, inflammatory reaction, and autophagy are significant contributors to the pathogenesis of OP. Besides, epigenetic changes, such as changes in non-coding RNA and DNA methylation, are also crucial in the pathogenesis of OP. Exercise-induced changes in mechanical stress, hormones, cytokines, epigenetics, and signaling pathways can regulate these pathological changes, thus regulating bone metabolism, thereby promoting bone formation. Different sports items, intensity, duration, and frequency may have varying effects on the body.

At present, there are few studies on the effects of exercise on circRNAs, DNA methylation, and autophagy in osteoporotic people. To investigate the mechanism of exercise to alleviate OP and to establish the foundation for clinical treatment, more clinical research as well as *in vitro* and *in vivo* trials are required.

## Author contributions

X-QW and HZ led the conception and design. LZ led the drafting and reviewing the manuscript. Y-LZ, RW were involved in the editing/revision process. All authors approved the final version and were involved in realization of this review.

## Funding

This work was supported by the National Natural Science Foundation of China (Fund Number: 81702666, 81872171, 81871844); the Shanghai Key Lab of Human Performance (Shanghai University of Sport) (11DZ2261100); Shanghai Frontiers Science Research Base of Exercise and Metabolic Health; Talent Development Fund of Shanghai Municipal (2021081); Shanghai Clinical Research Center for Rehabilitation Medicine (21MC1930200).

## Conflict of interest

The authors declare that the research was conducted in the absence of any commercial or financial relationships that could be construed as a potential conflict of interest.

The handling editor CH declared a shared parent affiliation with the author HZ at the time of review.

## Publisher’s note

All claims expressed in this article are solely those of the authors and do not necessarily represent those of their affiliated organizations, or those of the publisher, the editors and the reviewers. Any product that may be evaluated in this article, or claim that may be made by its manufacturer, is not guaranteed or endorsed by the publisher.

## References

[1] Johnell O Kanis JA. An estimate of the worldwide prevalence and disability associated with osteoporotic fractures. Osteoporosis Int (2006) 17(12):1726–33. doi: 10.1007/s00198-006-0172-4 16983459

[2] Johnston CB Dagar M. Osteoporosis in older adults. Med Clin N Am (2020) 104(5):873–84. doi: 10.1016/j.mcna.2020.06.004 32773051

[3] Chen X Zhu X Wei A Chen F Gao Q Lu K. Nrf2 epigenetic derepression induced by running exercise protects against osteoporosis. Bone Res (2021) 9(1):15. doi: 10.1038/s41413-020-00128-8 33637693 PMC7910611

[4] Eastell R. Treatment of postmenopausal osteoporosis. New Engl J Med (1998) 338(11):736–46. doi: 10.1056/NEJM199803123381107 9494151

[5] Mohammad Rahimi GR Smart NA Liang MTC Bijeh N Albanaqi AL Fathi M. The impact of different modes of exercise training on bone mineral density in older postmenopausal women: A systematic review and meta-analysis research. Calcified Tissue Int (2020) 106(6):577–90. doi: 10.1007/s00223-020-00671-w 32055889

[6] Ott SM. In osteoporosis or osteopenia, exercise interventions improve BMD; effects vary by exercise type and BMD site. Ann Intern Med (2022) 175(4):C46. doi: 10.7326/J22-0014 35377720

[7] Riggs BL. The mechanisms of estrogen regulation of bone resorption. J Clin Invest (2000) 106(10):1203–4. doi: 10.1172/JCI11468 PMC381441 11086020

[8] Fleisher TA. Apoptosis. Ann Allergy Asthma Immunol (1997) 78(3):245–249, 249-250. doi: 10.1016/S1081-1206(10)63176-6 9087147

[9] Weinstein RS Manolagas SC. Apoptosis and osteoporosis. Am J Med (2000) 108(2):153–64. doi: 10.1016/s0002-9343(99)00420-9 11126309

[10] Jilka RL Weinstein RS Parfitt AM Manolagas SC. Quantifying osteoblast and osteocyte apoptosis: Challenges and rewards. J Bone Miner Res (2007) 22(10):1492–501. doi: 10.1359/jbmr.070518 17542686

[11] Komori T. Cell death in chondrocytes, osteoblasts, and osteocytes. Int J Mol Sci (2016) 17(12):2045. doi: 10.3390/ijms17122045 PMC5187845 27929439

[12] Tanaka S. Molecular mechanism of the life and death of the osteoclast. Ann Ny Acad Sci (2006) 1068(1):180–6. doi: 10.1196/annals.1346.020 16831917

[13] Hughes DE Dai A Tiffee JC Li HH Mundy GR Boyce BF. Estrogen promotes apoptosis of murine osteoclasts mediated by TGF–β. Nat Med (1996) 2(10):1132–6. doi: 10.1038/nm1096-1132 8837613

[14] Espina B Liang M Russell RGG Hulley PA. Regulation of bim in glucocorticoid-mediated osteoblast apoptosis. J Cell Physiol (2008) 215(2):488–96. doi: 10.1002/jcp.21335 PMC2820732 18064628

[15] Xie H Tang L Luo X Wu X Wu X Zhou H. Suppressive effect of dexamethasone on TIMP-1 production involves murine osteoblastic MC3T3-E1 cell apoptosis. Amino Acids (2010) 38(4):1145–53. doi: 10.1007/s00726-009-0325-9 19629637

[16] Plotkin LI Manolagas SC Bellido T. Glucocorticoids induce osteocyte apoptosis by blocking focal adhesion kinase-mediated survival. Evidence for inside-out signaling leading to anoikis. J Biol Chem (2007) 282(33):24120–30. doi: 10.1074/jbc.M611435200 17581824

[17] Almeida M Han L Ambrogini E Weinstein RS Manolagas SC. Glucocorticoids and tumor necrosis factor α increase oxidative stress and suppress wnt protein signaling in osteoblasts. J Biol Chem (2011) 286(52):44326–35. doi: 10.1074/jbc.M111.283481 PMC3247937 22030390

[18] Aguirre JI Plotkin LI Stewart SA Weinstein RS Parfitt AM Manolagas SC. Osteocyte apoptosis is induced by weightlessness in mice and precedes osteoclast recruitment and bone loss. J Bone Miner Res (2006) 21(4):605–15. doi: 10.1359/jbmr.060107 16598381

[19] Davis HM Pacheco-Costa R Atkinson EG Brun LR Gortazar AR Harris J. Disruption of the Cx43/miR21 pathway leads to osteocyte apoptosis and increased osteoclastogenesis with aging. Aging Cell (2017) 16(3):551–63. doi: 10.1111/acel.12586 PMC5418188 28317237

[20] Cauley JA Danielson ME Boudreau RM Forrest KYZ Zmuda JM Pahor M. Inflammatory markers and incident fracture risk in older men and women: The health aging and body composition study. J Bone Miner Res (2007) 22(7):1088–95. doi: 10.1359/jbmr.070409 17419681

[21] Barbour KE Boudreau R Danielson ME Youk AO Wactawski-Wende J Greep NC. Inflammatory markers and the risk of hip fracture: The women's health initiative. J Bone Miner Res (2012) 27(5):1167–76. doi: 10.1002/jbmr.1559 PMC3361578 22392817

[22] Ding C Parameswaran V Udayan R Burgess J Jones G. Circulating levels of inflammatory markers predict change in bone mineral density and resorption in older adults: A longitudinal study. J Clin Endocrinol Metab (2008) 93(5):1952–8. doi: 10.1210/jc.2007-2325 18285417

[23] Fischer V Haffner-Luntzer M. Interaction between bone and immune cells: Implications for postmenopausal osteoporosis. Semin Cell Dev Biol (2022) 123:14–21. doi: 10.1016/j.semcdb.2021.05.014 34024716

[24] Manolagas SC. Birth and death of bone cells: Basic regulatory mechanisms and implications for the pathogenesis and treatment of osteoporosis*. Endocr Rev (2000) 21(2):115–37. doi: 10.1210/edrv.21.2.0395 10782361

[25] Pacifici R. Estrogen, cytokines, and pathogenesis of postmenopausal osteoporosis. J Bone Miner Res (1996) 11(8):1043–51. doi: 10.1002/jbmr.5650110802 8854239

[26] Riggs BL Khosla S Melton LJ. Sex steroids and the construction and conservation of the adult skeleton. Endocr Rev (2002) 23(3):279–302. doi: 10.1210/edrv.23.3.0465 12050121

[27] Clowes JA Riggs BL Khosla S. The role of the immune system in the pathophysiology of osteoporosis. Immunol Rev (2005) 208(1):207–27. doi: 10.1111/j.0105-2896.2005.00334.x 16313351

[28] Zha L He L Liang Y Qin H Yu B Chang L. TNF-α contributes to postmenopausal osteoporosis by synergistically promoting RANKL-induced osteoclast formation. Biomed Pharmacothe (2018) 102:369–74. doi: 10.1016/j.biopha.2018.03.080 29571022

[29] Hofbauer LC Khosla S Dunstan CR Lacey DL Boyle WJ Riggs BL. The roles of osteoprotegerin and osteoprotegerin ligand in the paracrine regulation of bone resorption. J Bone Miner Res (2000) 15(1):2–12. doi: 10.1359/jbmr.2000.15.1.2 10646108

[30] Terkawi MA Matsumae G Shimizu T Takahashi D Kadoya K Iwasaki N. Interplay between inflammation and pathological bone resorption: Insights into recent mechanisms and pathways in related diseases for future perspectives. Int J Mol Sci (2022) 23(3):1786. doi: 10.3390/ijms23031786 35163708 PMC8836472

[31] Breuil V Ticchioni M Testa J Roux CH Ferrari P Breittmayer JP. Immune changes in post-menopausal osteoporosis: The immunos study. Osteoporosis Int (2010) 21(5):805–14. doi: 10.1007/s00198-009-1018-7 19876583

[32] Pietschmann P Grisar J Thien R Willheim M Kerschan-Schindl K Preisinger E. Immune phenotype and intracellular cytokine production of peripheral blood mononuclear cells from postmenopausal patients with osteoporotic fractures. Exp Gerontol (2001) 36(10):1749–59. doi: 10.1016/s0531-5565(01)00125-5 11672994

[33] Abildgaard J Tingstedt J Zhao Y Hartling HJ Pedersen AT Lindegaard B. Increased systemic inflammation and altered distribution of T-cell subsets in postmenopausal women. PloS One (2020) 15(6):e235174. doi: 10.1371/journal.pone.0235174 PMC7310708 32574226

[34] Fang H Zhang H Wang Z Zhou Z Li Y Lu L. Systemic immune-inflammation index acts as a novel diagnostic biomarker for postmenopausal osteoporosis and could predict the risk of osteoporotic fracture. J Clin Lab Anal (2019) 34(1):e23016. doi: 10.1002/jcla.23016 31423643 PMC6977145

[35] Levine B Kroemer G. Autophagy in the pathogenesis of disease. Cell (2008) 132(1):27–42. doi: 10.1016/j.cell.2007.12.018 18191218 PMC2696814

[36] Deretic V Saitoh T Akira S. Autophagy in infection, inflammation and immunity. Nat Rev Immunol (2013) 13(10):722–37. doi: 10.1038/nri3532 PMC5340150 24064518

[37] Wang T He H Liu S Jia C Fan Z Zhong C. Autophagy: A promising target for age-related osteoporosis. Curr Drug Targets (2019) 20(3):354–65. doi: 10.2174/1389450119666180626120852 29943700

[38] Zhang L Guo Y Liu Y Liu Y Xiong D Liu X. Pathway-based genome-wide association analysis identified the importance of regulation-of-autophagy pathway for ultradistal radius BMD. J Bone Miner Res (2010) 25(7):1572–80. doi: 10.1002/jbmr.36 PMC3153999 20200951

[39] Chen K Yang Y Jiang S Jiang L. Decreased activity of osteocyte autophagy with aging may contribute to the bone loss in senile population. Histochem Cell Biol (2014) 142(3):285–95. doi: 10.1007/s00418-014-1194-1 24553790

[40] Wu JJ Quijano C Chen E Liu H Cao L Fergusson MM. Mitochondrial dysfunction and oxidative stress mediate the physiological impairment induced by the disruption of autophagy. Aging (2009) 1(4):425–37. doi: 10.18632/aging.100038 PMC2806022 20157526

[41] Høyer-Hansen M Nordbrandt SPS Jäättelä M. Autophagy as a basis for the health-promoting effects of vitamin d. Trends Mol Med (2010) 16(7):295–302. doi: 10.1016/j.molmed.2010.04.005 20488750

[42] Tang N Zhao H Zhang H Dong Y. Effect of autophagy geneDRAM on proliferation, cell cycle, apoptosis, and autophagy of osteoblast in osteoporosis rats. J Cell Physiol (2019) 234(4):5023–32. doi: 10.1002/jcp.27304 30203495

[43] Liu F Fang F Yuan H Yang D Chen Y Williams L. Suppression of autophagy by FIP200 deletion leads to osteopenia in mice through the inhibition of osteoblast terminal differentiation. J Bone Miner Res (2013) 28(11):2414–30. doi: 10.1002/jbmr.1971 PMC3805719 23633228

[44] Onal M Piemontese M Xiong J Wang Y Han L Ye S. Suppression of autophagy in osteocytes mimics skeletal aging. J Biol Chem (2013) 288(24):17432–40. doi: 10.1074/jbc.M112.444190 PMC3682543 23645674

[45] Li DY Yu JC Xiao L Miao W Ji K Wang SC. Autophagy attenuates the oxidative stress-induced apoptosis of Mc3T3-E1 osteoblasts. Eur Rev Med Pharmacol Sci (2017) 21(24):5548–56. doi: 10.26355/eurrev_201712_13991 29271985

[46] Liu Z Hong C Hu W Chen M Duan R Li H. Autophagy receptor OPTN (optineurin) regulates mesenchymal stem cell fate and bone-fat balance during aging by clearing FABP3. Autophagy (2021) 17(10):2766–82. doi: 10.1080/15548627.2020.1839286 PMC8526045 33143524

[47] Lin N Chen C Kagwiria R Liang R Beyer C Distler A. Inactivation of autophagy ameliorates glucocorticoid-induced and ovariectomy-induced bone loss. Ann Rheumatol Dis (2016) 75(6):1203–10. doi: 10.1136/annrheumdis-2015-207240 26113650

[48] Xiu Y Xu H Zhao C Li J Morita Y Yao Z. Chloroquine reduces osteoclastogenesis in murine osteoporosis by preventing TRAF3 degradation. J Clin Invest (2014) 124(1):297–310. doi: 10.1172/JCI66947 24316970 PMC3871219

[49] Jin D Wu X Yu H Jiang L Zhou P Yao X. Systematic analysis of lncRNAs, mRNAs, circRNAs and miRNAs in patients with postmenopausal osteoporosis. Am J Transl Res (2018) 10(5):1498–510. PMC5992556 29887963

[50] Cao J. The functional role of long non-coding RNAs and epigenetics. Biol Proced Online (2014) 16(1):11. doi: 10.1186/1480-9222-16-11 25276098 PMC4177375

[51] Wang Q Li Y Zhang Y Ma L Lin L Meng J. LncRNA MEG3 inhibited osteogenic differentiation of bone marrow mesenchymal stem cells from postmenopausal osteoporosis by targeting miR-133a-3p. Biomed Pharmacothe (2017) 89:1178–86. doi: 10.1016/j.biopha.2017.02.090 28320084

[52] Liu C Gao X Li Y Sun W Xu Y Tan Y. The mechanosensitive lncRNA Neat1 promotes osteoblast function through paraspeckle-dependent Smurf1 mRNA retention. Bone Res (2022) 10(1):18. doi: 10.1038/s41413-022-00191-3 35210394 PMC8873336

[53] Chen C Cheng P Xie H Zhou H Wu X Liao E. MiR-503 regulates osteoclastogenesis *via* targeting RANK. J Bone Miner Res (2014) 29(2):338–47. doi: 10.1002/jbmr.2032 23821519

[54] Sugatani T Vacher J Hruska KA. A microRNA expression signature of osteoclastogenesis. Blood (2011) 117(13):3648–57. doi: 10.1182/blood-2010-10-311415 PMC3072882 21273303

[55] Huang X Cen X Zhang B Liao Y Zhao Z Zhu G. The roles of circRFWD2 and circINO80 during NELL-1-induced osteogenesis. J Cell Mol Med (2019) 23(12):8432–41. doi: 10.1111/jcmm.14726 PMC6850935 31633307

[56] Ouyang Z Tan T Zhang X Wan J Zhou Y Jiang G. CircRNA hsa_circ_0074834 promotes the osteogenesis-angiogenesis coupling process in bone mesenchymal stem cells (BMSCs) by acting as a ceRNA for miR-942-5p. Cell Death Dis (2019) 10(12):932. doi: 10.1038/s41419-019-2161-5 31804461 PMC6895238

[57] McGee SL Hargreaves M. Epigenetics and exercise. Trends Endocrinol Metab (2019) 30(9):636–45. doi: 10.1016/j.tem.2019.06.002 31279665

[58] Moore LD Le T Fan G. DNA Methylation and its basic function. Neuropsychopharmacol (2013) 38(1):23–38. doi: 10.1038/npp.2012.112 PMC3521964 22781841

[59] Bird A. DNA Methylation patterns and epigenetic memory. Gene Dev (2002) 16(1):6–21. doi: 10.1101/gad.947102 11782440

[60] Marini F Cianferotti L Brandi M. Epigenetic mechanisms in bone biology and osteoporosis: Can they drive therapeutic choices? Int J Mol Sci (2016) 17(8):1329. doi: 10.3390/ijms17081329 PMC5000726 27529237

[61] Vrtačnik P Marc J Ostanek B. Epigenetic mechanisms in bone. Clin Chem Lab Med (2014) 52(5):589–608. doi: 10.1515/cclm-2013-0770 24353145

[62] Reppe S Lien TG Hsu Y Gautvik VT Olstad OK Yu R. Distinct DNA methylation profiles in bone and blood of osteoporotic and healthy postmenopausal women. Epigenetics-US (2017) 12(8):674–87. doi: 10.1080/15592294.2017.1345832 PMC5687328 28650214

[63] Raje MM Ashma R. Epigenetic regulation of BMP2 gene in osteoporosis: A DNA methylation study. Mol Biol Rep (2019) 46(2):1667–74. doi: 10.1007/s11033-019-04615-y 30788762

[64] Nishikawa K Iwamoto Y Kobayashi Y Katsuoka F Kawaguchi S Tsujita T. DNA Methyltransferase 3a regulates osteoclast differentiation by coupling to an s-adenosylmethionine–producing metabolic pathway. Nat Med (2015) 21(3):281–7. doi: 10.1038/nm.3774 25706873

[65] Moller A Delaisse JM Olesen JB Madsen JS Canto LM Bechmann T. Aging and menopause reprogram osteoclast precursors for aggressive bone resorption. Bone Res (2020) 8(1):27. doi: 10.1038/s41413-020-0102-7 32637185 PMC7329827

[66] Turner CH Robling AG. Designing exercise regimens to increase bone strength. Exerc Sport Sci Rev (2003) 31(1):45–50. doi: 10.1097/00003677-200301000-00009 12562170

[67] Chastin SFM Mandrichenko O Helbostadt JL Skelton DA. Associations between objectively-measured sedentary behaviour and physical activity with bone mineral density in adults and older adults, the NHANES study. Bone (New York N.Y.) (2014) 64:254–62. doi: 10.1016/j.bone.2014.04.009 24735973

[68] Sherk VD Rosen CJ. Senescent and apoptotic osteocytes and aging: Exercise to the rescue? Bone (2019) 121:255–8. doi: 10.1016/j.bone.2019.02.006 PMC6459182 30735796

[69] Robling AG Hinant FM Burr DB Turner CH. Improved bone structure and strength after long-term mechanical loading is greatest if loading is separated into short bouts. J Bone Miner Res (2002) 17(8):1545–54. doi: 10.1359/jbmr.2002.17.8.1545 12162508

[70] Rochefort GY Pallu S Benhamou CL. Osteocyte: The unrecognized side of bone tissue. Osteoporosis Int (2010) 21(9):1457–69. doi: 10.1007/s00198-010-1194-5 20204595

[71] Basat H Esmaeilzadeh S Eskiyurt N. The effects of strengthening and high-impact exercises on bone metabolism and quality of life in postmenopausal women: A randomized controlled trial. J Back Musculoskelet Rehabil (2013) 26(4):427–35. doi: 10.3233/BMR-130402 23948830

[72] Vélez-Toral M Godoy-Izquierdo D de Guevara NML de Teresa Galván C Ballesteros AS García JFG. Improvements in health-related quality of life, cardio-metabolic health, and fitness in postmenopausal women after an exercise plus health promotion intervention: A randomized controlled trial. J Phys Activity Health (2017) 14(5):336–43. doi: 10.1123/jpah.2016-0218 28169553

[73] Wen HJ Huang TH Li TL Chong PN Ang BS. Effects of short-term step aerobics exercise on bone metabolism and functional fitness in postmenopausal women with low bone mass. Osteoporosis Int (2017) 28(2):539–47. doi: 10.1007/s00198-016-3759-4 27613719

[74] Bonewald L. Use it or lose it to age: A review of bone and muscle communication. Bone (2019) 120:212–8. doi: 10.1016/j.bone.2018.11.002 PMC6360108 30408611

[75] Zhang D Bae C Lee J Lee J Jin Z Kang M. The bone anabolic effects of irisin are through preferential stimulation of aerobic glycolysis. Bone (2018) 114:150–60. doi: 10.1016/j.bone.2018.05.013 29775761

[76] Crane JD Devries MC Safdar A Hamadeh MJ Tarnopolsky MA. The effect of aging on human skeletal muscle mitochondrial and intramyocellular lipid ultrastructure. J Gerontol A Biol Sci Med Sci (2010) 65(2):119–28. doi: 10.1093/gerona/glp179 19959566

[77] Lanza IR Short DK Short KR Raghavakaimal S Basu R Joyner MJ. Endurance exercise as a countermeasure for aging. Diabetes (2008) 57(11):2933–42. doi: 10.2337/db08-0349 PMC2570389 18716044

[78] Lomas-Vega R Obrero-Gaitán E Molina-Ortega FJ Del-Pino-Casado R. Tai chi for risk of falls. A meta-analysis. J Am Geriatr Soc (2017) 65(9):2037–43. doi: 10.1111/jgs.15008 28736853

[79] Tiedemann A O'Rourke S Sesto R Sherrington C. A 12-week iyengar yoga program improved balance and mobility in older community-dwelling people: A pilot randomized controlled trial. J Gerontol A Biol Sci Med Sci (2013) 68(9):1068–75. doi: 10.1093/gerona/glt087 23825035

[80] Aveline P Cesaro A Mazor M Best TM Lespessailles E Toumi H. Cumulative effects of strontium ranelate and impact exercise on bone mass in ovariectomized rats. Int J Mol Sci (2021) 22(6):3040. doi: 10.3390/ijms22063040 33809778 PMC8002366

[81] Maurel DB Boisseau N Pallu S Rochefort GY Benhamou C Jaffre C. Regular exercise limits alcohol effects on trabecular, cortical thickness and porosity, and osteocyte apoptosis in the rat. Joint Bone Spine (2013) 80(5):492–8. doi: 10.1016/j.jbspin.2012.12.005 23380443

[82] Wen J Bao M Tang M He X Yao X Li L. Low magnitude vibration alleviates age-related bone loss by inhibiting cell senescence of osteogenic cells in naturally senescent rats. Aging (Albany NY.) (2021) 13(8):12031–45. doi: 10.18632/aging.202907 PMC8109117 33888646

[83] Li L Chen X Lv S Dong M Zhang L Tu J. Influence of exercise on bone remodeling-related hormones and cytokines in ovariectomized rats: A model of postmenopausal osteoporosis. PloS One (2014) 9(11):e112845. doi: 10.1371/journal.pone.0112845 25393283 PMC4231162

[84] Gao HE Wu DS Sun L Yang LD Qiao YB Ma S. Effects of lifelong exercise on age-related body composition, oxidative stress, inflammatory cytokines, and skeletal muscle proteome in rats. Mech Ageing Dev (2020) 189:111262. doi: 10.1016/j.mad.2020.111262 32422206

[85] Lee S Shin YA Cho J Park DH Kim C. Trabecular bone microarchitecture improvement is associated with skeletal nerve increase following aerobic exercise training in middle-aged mice. Front Physiol (2021) 12:800301. doi: 10.3389/fphys.2021.800301 35273515 PMC8902445

[86] Zuo B Zhu J Li J Wang C Zhao X Cai G. MicroRNA-103a functions as a mechanosensitive microRNA to inhibit bone formation through targeting Runx2. J Bone Miner Res (2015) 30(2):330–45. doi: 10.1002/jbmr.2352 25195535

[87] Filipović TN Lazović MP Backović AN Filipović AN Ignjatović AM Dimitrijević SS. A 12-week exercise program improves functional status in postmenopausal osteoporotic women: Randomized controlled study. Eur J Phys Rehab Med (2021) 57(1):120–30. doi: 10.23736/S1973-9087.20.06149-3 32902207

[88] Hettchen M von Stengel S Kohl M Murphy MH Shojaa M Ghasemikaram M. Changes in menopausal risk factors in early postmenopausal osteopenic women after 13 months of high-intensity exercise: The randomized controlled ACTLIFE-RCT. Clin Interv Aging Volume (2021) 16:83–96. doi: 10.2147/CIA.S283177 PMC7810823 33469276

[89] Kistler Fischbacher M Yong JS Weeks BK Beck BR. A comparison of bone-targeted exercise with and without antiresorptive bone medication to reduce indices of fracture risk in postmenopausal women with low bone mass: The MEDEX-OP randomized controlled trial. J Bone Miner Res (2021) 36(9):1680–93. doi: 10.1002/jbmr.4334 34033146

[90] Stanghelle B Bentzen H Giangregorio L Pripp AH Skelton DA Bergland A. Effects of a resistance and balance exercise programme on physical fitness, health-related quality of life and fear of falling in older women with osteoporosis and vertebral fracture: A randomized controlled trial. Osteoporosis Int (2020a) 31(6):1069–78. doi: 10.1007/s00198-019-05256-4 31925473

[91] Stanghelle B Bentzen H Giangregorio L Pripp AH Skelton DA Bergland A. Physical fitness in older women with osteoporosis and vertebral fracture after a resistance and balance exercise programme: 3-month post-intervention follow-up of a randomised controlled trial. BMC Musculoskel Dis (2020b) 21(1):471. doi: 10.1186/s12891-020-03495-9 PMC7368978 32682416

[92] Kemmler W Kohl M Fröhlich M Jakob F Engelke K Stengel S. Effects of high-intensity resistance training on osteopenia and sarcopenia parameters in older men with osteosarcopenia–one-year results of the randomized controlled franconian osteopenia and sarcopenia trial (FrOST ). J Bone Miner Res (2020) 35(9):1634–44. doi: 10.1002/jbmr.4027 32270891

[93] Harding AT Weeks BK Lambert C Watson SL Weis LJ Beck BR. A comparison of bone-targeted exercise strategies to reduce fracture risk in middle-aged and older men with osteopenia and osteoporosis:LIFTMOR-m semi-randomized controlled trial. J Bone Miner Res (2020) 35(8):1404–14. doi: 10.1002/jbmr.4008 32176813

[94] Filipović T Gopčević K Dimitrijević S Hrković M Backović A Lazović M. Effects of 12-week exercise program on enzyme activity of serum matrix metalloproteinase-9 and tissue inhibitor of metalloproteinase-1 in female patients with postmenopausal osteoporosis: A randomized control study. BioMed Res Int (2020) 2020:1–9. doi: 10.1155/2020/9758289 PMC7011433 32071923

[95] Harding AT Weeks BK Lambert C Watson SL Weis LJ Beck BR. Effects of supervised high-intensity resistance and impact training or machine-based isometric training on regional bone geometry and strength in middle-aged and older men with low bone mass: The LIFTMOR-m semi-randomised controlled trial. Bone (2020) 136:115362. doi: 10.1016/j.bone.2020.115362 32289518

[96] ElDeeb AM Abdel-Aziem AA. Effect of whole-body vibration exercise on power profile and bone mineral density in postmenopausal women with osteoporosis: A randomized controlled trial. J Manip Physiol Ther (2020) 43(4):384–93. doi: 10.1016/j.jmpt.2019.12.003 32868028

[97] Sen EI Esmaeilzadeh S Eskiyurt N. Effects of whole-body vibration and high impact exercises on the bone metabolism and functional mobility in postmenopausal women. J Bone Miner Metab (2020) 38(3):392–404. doi: 10.1007/s00774-019-01072-2 31897748

[98] Pérez-Gómez J Adsuar JC García-Gordillo MÁ. Muñoz P Romo L Maynar M. Twelve weeks of whole body vibration training improve regucalcin, body composition and physical fitness in postmenopausal women: A pilot study. Int J Env Res Pub He (2020) 17(11):3940. doi: 10.3390/ijerph17113940 PMC7312189 32498351

[99] Pasqualini L Ministrini S Lombardini R Bagaglia F Paltriccia R Pippi R. Effects of a 3-month weight-bearing and resistance exercise training on circulating osteogenic cells and bone formation markers in postmenopausal women with low bone mass. Osteoporosis Int (2019) 30(4):797–806. doi: 10.1007/s00198-019-04908-9 30809725

[100] Morelli C Avolio E Galluccio A Caparello G Manes E Ferraro S. Impact of vigorous-intensity physical activity on body composition parameters, lipid profile markers, and irisin levels in adolescents: A cross-sectional study. Nutrients (2020) 12(3):742. doi: 10.3390/nu12030742 PMC7146488 32168929

[101] Storlino G Colaianni G Sanesi L Lippo L Brunetti G Errede M. Irisin prevents disuse-induced osteocyte apoptosis. J Bone Miner Res (2020) 35(4):766–75. doi: 10.1002/jbmr.3944 31826311

[102] Xu L Shen L Yu X Li P Wang Q Li C. Effects of irisin on osteoblast apoptosis and osteoporosis in postmenopausal osteoporosis rats through upregulating Nrf2 and inhibiting NLRP3 inflammasome. Exp Ther Med (2019) 19(2):1084–90. doi: 10.3892/etm.2019.8313 PMC6966163 32010273

[103] Ishigami A Fujita T Handa S Shirasawa T Koseki H Kitamura T. Senescence marker protein-30 knockout mouse liver is highly susceptible to tumor necrosis factor-alpha- and fas-mediated apoptosis. Am J Pathol (2002) 161(4):1273–81. doi: 10.1016/s0002-9440(10)64404-5 PMC1867294 12368201

[104] Fujita T Uchida K Maruyama N. Purification of senescence marker protein-30 (SMP30) and its androgen-independent decrease with age in the rat liver. Biochim Biophys Acta (1992) 1116(2):122–8. doi: 10.1016/0304-4165(92)90108-7 1581340

[105] Yuan Y Chen X Zhang L Wu J Guo J Zou D. The roles of exercise in bone remodeling and in prevention and treatment of osteoporosis. Prog Biophys Mol Biol (2016) 122(2):122–30. doi: 10.1016/j.pbiomolbio.2015.11.005 26657214

[106] Cabral-Santos C de Lima JE Fernandes I Pinto RZ Rosa-Neto JC Bishop NC. Interleukin-10 responses from acute exercise in healthy subjects: A systematic review. J Cell Physiol (2019) 234(7):9956–65. doi: 10.1002/jcp.27920 30536945

[107] Santos RV Viana VA Boscolo RA Marques VG Santana MG Lira FS. Moderate exercise training modulates cytokine profile and sleep in elderly people. Cytokine (2012) 60(3):731–5. doi: 10.1016/j.cyto.2012.07.028 22917967

[108] Monteiro-Junior RS de Tarso Maciel-Pinheiro P Da Matta Mello Portugal E Da Silva Figueiredo LF Terra R Carneiro LSF. Effect of exercise on inflammatory profile of older persons: Systematic review and meta-analyses. J Phys Activity Health (2018) 15(1):64–71. doi: 10.1123/jpah.2016-0735 28771081

[109] Stewart LK Flynn MG Campbell WW Craig BA Robinson JP McFarlin BK. Influence of exercise training and age on CD14+ cell-surface expression of toll-like receptor 2 and 4. Brain Behavior Immun (2005) 19(5):389–97. doi: 10.1016/j.bbi.2005.04.003 15963685

[110] Dalle CL Mottes M Cheri S Deiana M Zamboni F Gabbiani D. Increased gene expression of RUNX2 and SOX9 in mesenchymal circulating progenitors is associated with autophagy during physical activity. Oxid Med Cell Longev (2019) 2019:8426259. doi: 10.1155/2019/8426259 31737174 PMC6815530

[111] Mizushima N Yoshimori T. How to interpret LC3 immunoblotting. Autophagy (2007) 3(6):542–5. doi: 10.4161/auto.4600 17611390

[112] Zhang B Hou R Zou Z Luo T Zhang Y Wang L. Mechanically induced autophagy is associated with ATP metabolism and cellular viability in osteocytes *in vitro* . Redox Biol (2018) 14:492–8. doi: 10.1016/j.redox.2017.10.021 PMC5680519 29096322

[113] Kim SH Ko IG Jin JJ Hwang L Yoon HS Baek SS. Treadmill exercise ameliorates ethanol with lipopolysaccharide and carbon tetrachloride-mediated liver injury in mice. J Exerc Rehabil (2022) 18(1):28–33. doi: 10.12965/jer.2244002.001 35356144 PMC8934608

[114] Pantovic A Krstic A Janjetovic K Kocic J Harhaji-Trajkovic L Bugarski D. Coordinated time-dependent modulation of AMPK/Akt/mTOR signaling and autophagy controls osteogenic differentiation of human mesenchymal stem cells. Bone (2013) 52(1):524–31. doi: 10.1016/j.bone.2012.10.024 23111315

[115] Chen X Sun K Zhao S Geng T Fan X Sun S. Irisin promotes osteogenic differentiation of bone marrow mesenchymal stem cells by activating autophagy *via* the wnt//β-catenin signal pathway. Cytokine (2020) 136:155292. doi: 10.1016/j.cyto.2020.155292 32950809

[116] An T He ZC Zhang XQ Li J Chen AL Tan F. Baduanjin exerts anti-diabetic and anti-depression effects by regulating the expression of mRNA, lncRNA, and circRNA. Chin Med (2019) 14:3. doi: 10.1186/s13020-019-0225-1 30733823 PMC6359771

[117] Zhu G Zeng C Qian Y Yuan S Ye Z Zhao S. Tensile strain promotes osteogenic differentiation of bone marrow mesenchymal stem cells through upregulating lncRNA-MEG3. Histol Histopathol (2021) 36(9):939–46. doi: 10.14670/HH-18-365 34318924

[118] Ntanasis-Stathopoulos J Tzanninis JG Philippou A Koutsilieris M. Epigenetic regulation on gene expression induced by physical exercise. J Musculoskelet Neuronal Interact (2013) 13(2):133–46. 23728100

[119] Guo M Qiu J Shen F Wang S Yu J Zuo H. Comprehensive analysis of circular RNA profiles in skeletal muscles of aging mice and after aerobic exercise intervention. Aging (Albany NY) (2020) 12(6):5071–90. doi: 10.18632/aging.102932 PMC7138574 32182212

[120] Fang L Lin L Lv Y Huang Z Lin X Wang X. The mechanism of aerobic exercise combined with glucosamine therapy and circUNK in improving knee osteoarthritis in rabbits. Life Sci (2021) 275:119375. doi: 10.1016/j.lfs.2021.119375 33737085

[121] Ferioli M Zauli G Maiorano P Milani D Mirandola P Neri LM. Role of physical exercise in the regulation of epigenetic mechanisms in inflammation, cancer, neurodegenerative diseases, and aging process. J Cell Physiol (2019) 234(9):14852–64. doi: 10.1002/jcp.28304 30767204

[122] Lindholm ME Marabita F Gomez-Cabrero D Rundqvist H Ekström TJ Tegnér J. An integrative analysis reveals coordinated reprogramming of the epigenome and the transcriptome in human skeletal muscle after training. Epigenetics-US (2014) 9(12):1557–69. doi: 10.4161/15592294.2014.982445 PMC4622000 25484259

[123] Rönn T Volkov P Davegårdh C Dayeh T Hall E Olsson AH. A six months exercise intervention influences the genome-wide DNA methylation pattern in human adipose tissue. PloS Genet (2013) 9(6):e1003572. doi: 10.1371/journal.pgen.1003572 23825961 PMC3694844

[124] Arnsdorf EJ Tummala P Castillo AB Zhang F Jacobs CR. The epigenetic mechanism of mechanically induced osteogenic differentiation. J Biomech (2010) 43(15):2881–6. doi: 10.1016/j.jbiomech.2010.07.033 PMC2975768 20728889

[125] Nakajima K Takeoka M Mori M Hashimoto S Sakurai A Nose H. Exercise effects on methylation of ASC gene. Int J Sports Med (2010) 31(9):671–5. doi: 10.1055/s-0029-1246140 20200803

